# Cut-off values for IL-21 and IL-23 as biochemical markers for pemphigus vulgaris

**DOI:** 10.25122/jml-2023-0226

**Published:** 2023-09

**Authors:** Zahra Ali Al-Hasnawi, Ban AL-Drobie

**Affiliations:** 1 Department of Oral Medicine, College of Dentistry, University of Baghdad, Baghdad, Iraq; 2 Department of Oral Diagnosis, College of Dentistry, University of Baghdad, Baghdad, Iraq

**Keywords:** Pemphigus vulgaris, IL-21, IL-23, cuttoff point

## Abstract

Pemphigus vulgaris (PV) is a potentially fatal mucocutaneous autoimmune disease characterized by severe skin lesions. Interleukin-21 (IL-21) and IL-23 have been linked to several autoimmune inflammatory diseases that may have a critical role in PV immunopathogenesis, including T-helper 17 (Th17) development. This study aimed to compare the serum levels of IL-21 and IL-23 in patients with PV and healthy controls. This case-control study included 90 participants (45 patients and 45 controls). Serum IL-21 and IL-23 were measured using the Sandwich-ELISA method provided by Sunlong Biotech, China. The findings revealed statistically significant results for IL-21 O.D. and Conc. (p=0.012*) and highly significant results for IL-23 O.D. and Conc. (p=0.000**). Furthermore, cut-off values were established for IL-21 (O.D.=0.071 pg/mL, Conc.=6.468 pg/mL) and IL-23 (O.D.=0.141 pg/mL, Conc.=6.745 pg/mL). These results indicate a potential association between PV and IL-21, IL-23, and the identified cut-off values. The particular roles of cytokines and how they can be utilized to treat PV require more investigation. To our knowledge, this was the first study to detect a cut-off point for IL-21 and IL-23 that may be used as novel and cost-effective biochemical markers for disease diagnosis.

## INTRODUCTION

Pemphigus vulgaris (PV) is a severe mucocutaneous autoimmune disease that causes blisters at the mucous membranes and skin due to inadequate keratinocyte adhesion. Autoantibodies targeting desmoglein 3 and desmoglein 1 (Dsg3 and Dsg1) in the blood of PV patients lead to acantholysis, preventing keratinocytes from adhering to each other. These autoantibodies play a crucial role in the pathophysiology of PV, often leading to the rapid transformation of blisters into ulcers or bleeding erosions within 24 hours of eruption [1]. Globally, PV has an incidence ranging from 0.07 to 1.6 per 100,000 individuals and is associated with a mortality rate over 3.5 times higher than the general population. Sepsis and pneumonia are the most common causes of infection-related mortality. The patient is at risk of dying if they have inadequate therapy or a delayed diagnosis [2].

The clinical and histopathological diagnosis of PV involves skin or oral mucosal biopsies, typically requiring two samples: one for histological examination and the other for direct immunofluorescence (DIF). Ideally, a fresh vesicle less than 24 hours old is preferred for histopathology, but biopsies should be taken from the spreading periphery of the lesion due to the rarity of complete blisters [3].

Oral lesions are often the initial manifestation of PV, occurring in 80%-90% of patients at some point during the disease. In approximately 60% of cases, oral lesions are the first symptom. These oral lesions may last for weeks or even months before cutaneous lesions develop. In a minority of people, the oral mucosa is the only affected part, beginning with many superficial and irregular erosions. These erosions result from the detachment of a thin layer of epithelium with an abnormal appearance, exposing an erythematous (red) underlying tissue. Over several weeks, the lesion's border may gradually expand peripherally, potentially causing extensive damage to the oral mucosa. Lesions often start in the buccal mucosa along the occlusal plane, with gingiva, palate mucosa, and tongue commonly implicated. Lesions develop a more defined edge as they start to heal and finally have a white appearance before completely disappearing. Ulcers on the dorsum of the tongue can be long-lasting and may lead to loss of papillae [4].

The precise pathogenesis of PV remains incompletely understood, although B cell-produced autoantibodies against Dsg1 and Dsg3 are considered the primary cause [5]. In the first immunological processes, autoreactive T cells seem essential for developing autoantibodies in addition to B cells. These cells are sources of many cytokines that may work in an autocrine way and influence other immune cells from the innate/adaptive immune systems [6].

Interleukin (IL)-21 is primarily produced by natural killer T, T follicular helper (Tfh), and T-helper 17 (Th17) cells. This cytokine exhibits pleiotropic properties with specific effects on T and B cells [7]. It is produced by Th17 and Tfh cells and promotes various immune responses, including the synthesis of antibodies by Tfh cells and the development, activation, and differentiation of B cells. These processes produce plasma cells that generate antibodies and memory B cells, which contribute to the immune response [8]. The properties of IL-21 suggest its involvement in various immune system disorders, including pemphigus [9]. IL-23 has been associated with several autoimmune inflammatory illnesses, such as colitis, psoriasis, and arthritis. It is produced by dendritic cells and macrophages and causes an increase in the release of IL-17. Because of its ability to produce IL-17, IL-23 has a specific role in initiating and maintaining autoimmune inflammation [10]. Numerous studies revealed that the development of Th17 is the first step in expanding autoimmune activities involving IL-23. IL-23 is associated with Th17 formation and activity in patients with PV and may have an important role in PV immunopathogenesis, among other things [11]. This study compared serum IL-21 and IL-23 levels in patients with PV with healthy controls. As far as we know, this study represents the first attempt to establish a diagnostic cut-off point for these markers.

## MATERIAL AND METHODS

### Study design and participants

This case–control study was conducted between May and December 2022 at the Dermatology Department at Baghdad Teaching Hospital. Ninety individuals participated in this study, 45 of whom had PV based on clinical evaluation and histopathologic biopsy results. The control group consisted of 45 healthy individuals who did not exhibit any symptoms of cancer, autoimmune diseases, extensive inflammation, infectious diseases, or allergies. The age and sex of the two groups were matched.

### Blood collection

3 mL of venous blood was collected from each participant. Blood samples were allowed to clot at room temperature for 2 hours before being centrifuged for 5 minutes at 4000 revolutions per minute (rpm). Disposable, nonpyrogenic, and nonendotoxic blood collection tubes were used. Serum samples were then frozen at -40°C in polyethylene tubes until they were ready for assessment [12].

### Measurement of IL-21 and IL-23

Human IL-21 and IL-23 levels in the serum samples were determined using the sandwich ELISA (Enzyme-Linked Immunosorbent Assay) principle. ELISA kits from Sunlong Biotech/China [13] were employed.

### Statistical analysis

Statistical analysis was performed using SPSS version 22.0 and Microsoft Office Excel. Descriptive statistics were calculated, including observed percentages, frequencies, means, standard errors, standard deviations, and 95% confidence intervals. These calculations assumed a normal distribution of the data. Visual representations of data were created using stem–leaf plots, bar charts, and receiver operating characteristic (ROC) curve charts. The inferential analysis included the one-sample Kolmogorov–Smirnov test (K-S) to assess data distribution, contingency coefficients (C.C.) [14] to measure associations, and Student t-tests to compare means between groups [15]. Receiver operating characteristic (ROC) curve analysis determined the area under the curve (AUC) values, and cut-off points were established by estimating the small angular distance from the curve. Sensitivity and specificity testing helped identify the optimal cut-off point for discriminating between different study groups.

## RESULTS

Table 1 presents the distribution of socio-demographic characteristics variables (age and gender) and their significance in testing the assumption of random distribution in the studied groups. The total number of PV cases in the current study was 45. The age of PV patients ranged from 27 to 67 years, and the mean age was 45.36±10.60 years, while it was 37.02±10.49 years for the control group. The gender distribution in the patient and control groups was equal, with 31 (68.9%) females and 14 (31.1%) males in each group. The number of females was approximately twice as large as that of males in both groups. There was no statistical difference between the two groups (χ^2^=0.000, p=1.000, NS).

**Table 1:** Distribution of sociodemographic characteristics

Characteristics	Groups	PV Group		Control		C.S. (*) p-value
	Classes	No.	%	No.	%	
Age groups	20 -	5	11.1	9	20	χ^2^= 9.394 p=0.052 (NS)
	30 -	6	13.3	15	33.3	
	40 -	19	42.2	15	33.3	
	50 -	9	20.0	4	8.9	
	60 -70	6	13.3	2	4.4	
	Total	45	100	45	100	
	Mean±SD	45.36±10.60		37.02±10.49		
Gender	Male	14	31.1	14	31.1	χ^2^= 0.000 p=1.000 (NS)
	Female	31	68.9	31	68.9	
	Total	45	100	45	100	
C.S. (*) p-value		χ^2^= 3.411 p=0.492** (HS)		χ^2^= 3.007 p=0.557 (NS)		

CS: Computer Statistic; NS: Non-Significant (p>0.05); HS: Highly Significant (p≤0.01). Testing was conducted using Chi-Square and Two-Sample Chi-Square tests (χ^2^). Other abbreviations: SD: Standard Deviation; No: Number; %: Percent

The disease duration and site of lesions of the patient group are presented in Table 2. A significant proportion of the patients, approximately 68.9%, had a disease duration ranging from 1 to 10 years. The remaining cases were evenly divided into the two other categories, each accounting for approximately 15.6% of the total, with highly significant results among the three groups p=0.000** (HS), χ^2^=25.600.

**Table 2:** Distribution of history factors (disease duration, site of lesions)

History Factors	Classes	No.=45	%	C.S. ^(*)^ p-value
Disease duration yrs.	<1	7	15.6%	χ^2^= 25.600 p=0.000 (HS)
	1 _ 10	31	68.9%	
	>10	7	15.6%	
	Mean±SD	4.311±3.617		
Site of lesions	Mucocutaneous	30	66%	χ2= 28.689 p=0.000 (HS)
	Mucous	11	24.4%	
	Cutaneous	4	8.9%	

C.S. (*): Computer Statistic; Testing based on one Sample Chi-Square, and Binomial tests, HS (**): Highly significant at ** p≤ 0.01; y: year: No: number; %: percent

Most patients (66%) had mucocutaneous lesions, while mucous lesions were found in 24.4% of patients, and cutaneous lesions were identified in 8.9%. A highly significant difference was observed among the three groups, with a chi-square value of 28.689 and a p-value of 0.000**.

We conducted a goodness of fit test to assess the normal distribution assumption of IL-21 and IL-23, including optical density (O.D.) and concentration (Conc.) (Table 3). This test compared the observed cumulative distribution function with a theoretical normal distribution. The results indicated that these markers exhibited a normal distribution within the studied groups, as all p-values were greater than 0.05, indicating non-significance. This allowed us to apply standard statistical methods for descriptive and inferential analysis, assuming a normal data distribution.

**Table 3:** Distribution assessment for interleukin marker normality

**One-sample Kolmogorov–Smirnov test**					
Groups	Test statistic	IL-21 O.D.	IL-21 Conc.	IL-23 O.D.	IL-23 Conc.
Patients	No.	45	45	45	45
	Kolmogorov–Smirnov Z	1.105	1.105	1.175	1.168
	Asymp. sig. (two-tailed)	0.174	0.174	0.126	0.131
	C.S. ^(*)^	NS	NS	NS	NS
**Test distribution of data follows a normal shape**					
Control	No.	45	45	45	45
	Kolmogorov–Smirnov Z	0.864	0.864	0.718	0.840
	Asymp. sig. (two-tailed)	0.444	0.444	0.680	0.480
	C.S. ^(*)^	NS	NS	NS	NS
**Test distribution of data follows a normal shape**					

C.S. (*): Computer Statistic; IL: Interleukin; NS: Non-Sig. at p>0.05; No: number; K-S: Kolmogorov-Smirnov test; O.D.: optical density; Conc.: concentration

Table 4 provides a summary of statistics for the studied groups, including mean values, standard deviation, standard error, 95% confidence intervals (CI) for the mean, and the minimum and maximum values for IL21 and IL23, measured in terms of optical density (O.D.) and concentration (Conc). The results indicate that the mean values of interleukin markers were generally lower in the patient group, whether considering optical density or concentration markers, as well as standard deviation and standard error estimators. In addition, 95% confidence intervals indicate a decrease in the range of the intervals related to the estimation of the sampling population mean values for the patients group compared to the control. In addition, the corresponding intervals did not overlap between the two groups, in particular for IL23, in terms of optical density or concentration marker.

**Table 4:** Summary statistics of interleukin marker readings

Interleukin markers	Groups	No.	Mean	Std. D.	Std. E.	95% CI for mean		Min.	Max.
						L.b.	U.b.		
IL-21 O.D.	Patients	45	0.096	0.032	0.005	0.086	0.105	0.033	0.141
	Control	45	0.114	0.036	0.005	0.103	0.125	0.065	0.237
IL-21 Conc.	Patients	45	8.763	2.916	0.435	7.887	9.639	3.028	12.936
	Control	45	10.465	3.346	0.499	9.460	11.470	5.963	21.73
IL-23 O.D.	Patients	45	0.154	0.037	0.005	0.143	0.165	0.069	0.225
	Control	45	0.192	0.037	0.006	0.181	0.203	0.099	0.276
IL-23 Conc.	Patients	45	7.363	1.739	0.259	6.841	7.885	3.300	10.770
	Control	45	9.246	1.846	0.275	8.691	9.801	4.740	13.210

O.D.: Optical Density, Conc.: Concentration, L.b.: Lower bond, Ub: Upper bond, Std. D.: standard deviation, Std. E.: standard error

To test the compound statistical hypothesis, which assesses whether the studied groups, particularly in terms of interleukin markers, can be considered to originate from the same population, indicating similarity in readings between the two groups, we conducted t-tests for equality in variances and mean values (Table 5). As shown in Table 5, the results reveal significant findings for IL21 O.D. and Conc. (p=0.012*) and highly significant results for IL23 O.D. and Conc. (p=0.000**).

**Table 5:** Equal variances and mean values for interleukin marker tests among studied groups

Parameters	Levene’s test for equality of variances		t-test for equality of means	
	Levene’s statistic	Sig. ^(*)^	t-test	Sig. ^(*)^
IL-21 O.D.	0.020	0.888	−2.573	p=0.012* (S)
IL-21 Conc.	0.020	0.888	−2.573	p=0.012* (S)
IL-23 O.D.	0.654	0.421	−4.933	p=0.000** (HS)
IL-23 Conc.	0.214	0.645	−4.981	p=0.000** (HS)

(**) Highly significant at p<0.01; (*) significant at p<0.05. Testing is based on the compound statistical hypothesis: “equal variances and mean values are assumed.”

Table 6 presents the ROC curves, which allow for the visualization and selection of an ideal threshold that ensures the maximum level of decision accuracy. In this study, the analysis revealed that the area under the ROC curve for IL-21 was 0.610%, with a population value falling within the range of 0.492%–0.727%. Sensitivity and specificity were defined as 0.978% and 0.267%. The established IL-21 cut-off values for O.D. and Conc. were 0.071 and 6.468 pg/mL, respectively, with a 95% confidence interval of 0.492% to 0.727%. For IL-23, the area under the ROC curve was 0.768%, and its population value ranged from 0.670% to 0.865%. Its sensitivity and specificity were 0.933% and 0.378%, respectively. The IL-23 cut-off for O.D. and Conc. was established as 0.141 and 6.745 pg/mL, respectively, with 95% CI 0.670–0.865 for both O.D. and Conc.

**Table 6:** ROC analysis results for interleukin marker cut-off values and diagnostic performance

Markers	Cut-off point	Sen.	Spec.	Area	Std. error	Asymp. sig.	Asymp. 95% CI	
							L.b.	U.b.
IL-21 O.D.	0.071	0.978	0.267	0.610	0.060	0.073	0.492	0.727
IL-21 Conc.	6.468	0.978	0.267	0.610	0.060	0.073	0.492	0.727
IL-23 O.D.	0.141	0.933	0.378	0.768	0.050	0.000	0.670	0.865
IL-23 Conc.	6.745	0.933	0.378	0.768	0.050	0.000	0.670	0.865

(*) Highly significant at p<0.01; significant at p<0.05; nonsignificant at p>0.05. The positive actual state is positive.

In the subsequent analyses, IL-21 had no statistically significant findings with a p-value slightly above 0.05 (p=0.073). However, it is worth noting this result due to its potential relevance [16]. In contrast, IL-23 displayed highly significant results (p=0.000**). The ROC curve analysis demonstrated an area under the curve (AUC) of 0.865, suggesting its potential diagnostic value in the studied population. Figure 2 shows the graphical ROC curve plots for the studied markers.

**Figure 1:**
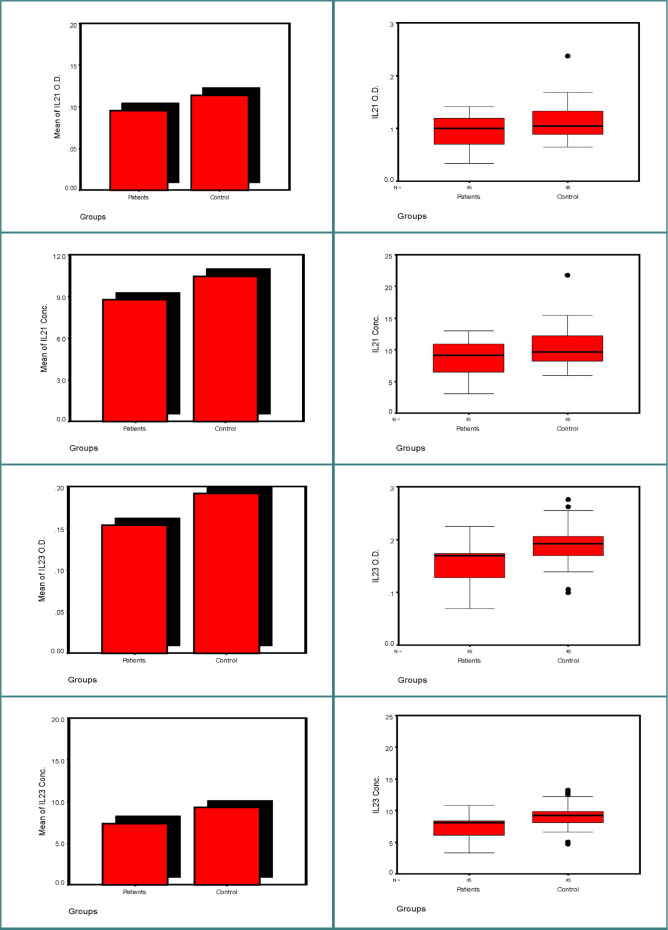
Bar charts for mean values and stem–leaf plots for interleukin marker readings among groups

**Figure 2:**
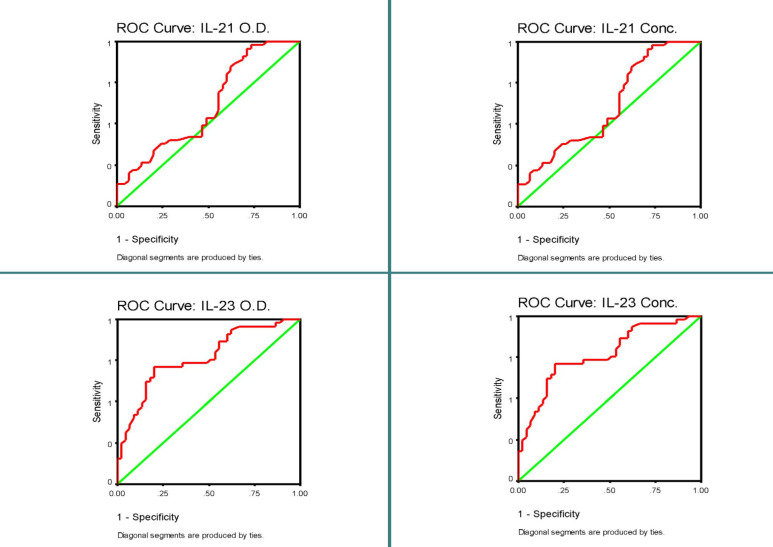
ROC curve plots for the studied markers comparing the control and patient group

## DISCUSSION

The statistical results of the study revealed a cut-off point and a direct association between IL-21, IL-23, and PV. This study explored the interactions between these interleukins and PV, potentially providing a unique and cost-efficient biochemical marker for diagnosing the disease. Because PV is a rare autoimmune illness and the research sample was small, this study has several limitations. Also, prolonged exposure to conventional immune-suppressing therapies could influence the study's outcomes [17].

The age of patients ranged from 27 to 67 years, and no statistically significant difference was observed between the age groups (χ^2^=9.394, p=0.052, NS). The data displayed a right-skewed distribution, with the mean age around 45.36±10.60 years. The control group displayed a trend of decreasing ages, consistent with findings from previous research conducted in Iraq [1] and China [18], where the average age fell within the range of 41 to 44 years. These results contrast other findings [19], which suggested an average age range of 50 to 55 years. Similar results were also reported in studies conducted in Japan [20]. The results could be due to a difference in sample size and ethnic variations.

The distribution of gender in both the patient and control groups was not similar, with a higher representation of females than males. In both groups, females constituted 68.9%, while males accounted for 31.1%. This indicates that there were approximately twice as many females as males in both groups, although there was no statistically significant difference in gender distribution between the two groups. This study aligns with several prior investigations that have reported a higher susceptibility of females to PV compared to males, consistent with findings in Iraq [4] and Morocco [21]. The increased prevalence of autoimmune diseases among females can be attributed to hormonal factors such as estrogens and progesterone and immunological and genetic factors, including specific genes on the X chromosome. These factors are believed to influence susceptibility to autoimmune diseases, potentially leading to more severe disease activity during pregnancy and postpartum relapses. The estrogen hormone may play a major role in increasing PV incidence among females since this hormone has been reported to enhance immunological reactions [22]. Other studies suggested that estrogen may compromise the cellular adhesion apparatus [23]. Contrary to our findings, some studies have indicated that males have higher PV incidence than females [24].

In the patient group of this study, the disease duration exhibited a distinct pattern, with more than two-thirds of the studied patients (68.9%) having a disease duration falling within the range of 1 to 10 years. The remaining cases were evenly distributed on both sides of this distribution, each accounting for 15.6% of the total. Disease duration, measured from the time of diagnosis, spanned from 0.5 to 15 years, a finding consistent with prior research conducted in Iraq [4] and Morocco [25]. This pattern may be attributed to the fact that a definitive diagnosis of pemphigus vulgaris is typically established after the first year. Additionally, the aggressive nature and high mortality rate associated with this disease make it rare for patients to have a disease duration exceeding 15 years.

The sites of PV lesions in the patient group (mucous, cutaneous, mucocutaneous) were significantly different. More than half of the patients had mucocutaneous lesions, accounting for 66.7% of the cases. This finding aligns with a study conducted in India [26], which also reported highly significant differences between the three lesion types, with mucocutaneous lesions being the most prevalent, constituting 92% of the cases. Similarly, a study in China [27] found highly significant differences between the three lesion types, with mucocutaneous lesions representing 55.5% of the patients. However, this result disagrees with a study in Iraq [4], where mucocutaneous PV accounted for less than a quarter of cases at the time of diagnosis, while most individuals had mucous PV lesions. The principal target antigens at cellular junctions in PV are Dsg-1 and Dsg-3, expressed in the skin and mucosal tissue. The distribution of the two proteins varies in different epithelia, such that in the skin, there is a high-level expression of Dsg-1 throughout the epidermis. In mucosal tissue, Dsg-3 is expressed across all layers. In PV, where anti-Dsg 3 antibodies predominate, there is generally marked mucocutaneous blistering [28].

Regarding the IL-21 result, a strong association exists between patients with PV and controls. The results of this study are similar to those of previous studies in Germany [29], China [27], and Egypt [30]. However, this outcome does not agree with a study in Iran [31], which found a nonsignificant relation between patients with PV and healthy controls in a genetic study. Patients with PV experience distinctive blistering and erosion as symptoms of the disease. After CD20+ B cell depletion, the majority of individuals with this disease have severe symptoms because they have autoantibodies against Dsg1 and Dsg3. Autoantibody synthesis is a crucial factor in the development of the illness [32]. Similarly, T cells are crucial for their interactions with B cells. However, the capacity of Tfh cells to enter Goblet cells (GCs) and continue generating B cell autoantibodies drew significant attention [33]. In a study by Hennerici *et al*., increased frequencies of Tfh cells, elevated IL-21 levels in plasma, and the presence of IL-21+ Dsg3 autoreactive T cells were identified in pemphigus patients with active disease. Circulating Tfh17 and Tfh17 were significantly increased in these patients, and these two Tfh cell populations were the major sources of IL-21. Research on skin lesions also demonstrated similar findings [29]. Additionally, the research revealed that the majority of invading T cells in skin lesions produce IL-21 and IL-17. Furthermore, resident memory T cells similar to Tfh mainly express IL-17 and IL-21. These results suggest that T-dependent immune responses and IL-21 are involved in pemphigus [34].

Regarding the IL-23 results, a highly significant relationship was observed between patients with PV and the control group. This finding aligns with the results of other studies conducted in China [35], Brazil [36], and Iran [37]. However, it contradicts the findings of a study conducted in Iran [38], which did not find a significant association between patients with PV and healthy controls. IL-23, a cytokine belonging to the IL-12 family, stimulates the inflammatory responses of Th17. Macrophages and dendritic cells were the main producers of IL-23 in the dermis. In the cutaneous lesions of patients with pemphigus, IL-23 levels increase. The role of IL-23 in autoimmune disease is exceedingly complicated because of the compound network of cytokines and immune cells, as well as the numerous impacts of IL-23/IL-17 [39]. The blood of patients with PV had higher levels of cytokines IFN- and IL-17, which are related to Th1 and Th17 immune responses, respectively. The increased levels of IFN- and IL-17 provide evidence that PV induces a mixed Th1/Th17 immune response. Th17 cell growth, which may have been encouraged by increased IL-23 production, was probably positive for PV pathogenesis [40].

Finally, we established a threshold for IL-21 and IL-23 levels to enhance the diagnostic method and identify the early stages of PV. The ROC curve analysis is a statistical technique to determine optimal cut-off values for the investigated parameters [41]. The IL-21 cut-off values for O.D. and Conc. were identified as 0.071 and 6.468 pg/mL, respectively. Similarly, the IL-23 cut-off values for O.D. and Conc. were determined as 0.141 and 6.745 pg/mL, respectively. To the best of our knowledge, this represents the first instance of establishing cut-off values for interleukins in the context of pemphigus vulgaris disease. The results indicate that IL-23 exhibited a highly significant area with a p-value of <0.001 when compared to the control group, whether considering optical density or concentration indicators. This suggests that IL-23 could serve as an excellent diagnostic marker for the studied disease, with an estimated area under the curve reaching up to 0.865, as supported by the 95% confidence interval. On the other hand, IL-21 did not show a significant area at p>0.05. However, it achieved a significance level of p=0.073. This significance level should be reported to provide a comprehensive understanding of the results rather than simply stating that statistical significance was not attained [16].

## CONCLUSION

Our study revealed a significant association between PV and the levels of IL-21 and IL-23, and we established distinct cut-off points for these cytokines. However, a comprehensive understanding of the particular roles of these cytokines and how they can be utilized to treat PV requires further investigation. As far as we know, this was the first study that detected a cut-off point for IL-21 and IL-23, which may be used as novel and cost-effective biochemical markers for disease diagnosis. Further research involving larger cohorts of patients with PV is needed to establish the relevance of IL-21 and IL-23 as reliable markers for diagnosing PV.
